# The global scientific consensus on genetically modified crop risk assessments

**DOI:** 10.1080/21645698.2026.2704430

**Published:** 2026-08-10

**Authors:** Luiza Favaratto, Savannah Gleim, Stuart J. Smyth

**Affiliations:** Department of Agricultural and Resource Economics, University of Saskatchewan, Saskatoon, Canada

**Keywords:** Barriers to innovation, biotechnology regulation, herbicide tolerance, insect resistance, regulatory barriers

## Abstract

Initial risk assessments of genetically modified (GM) crops began in the early 1990 s, focusing on herbicide tolerance and insect resistance, with approvals by the mid-1990s. These assessments compare GM crops to non-GM equivalents, a framework unchanged for over 30 years. Since the first assessment in 1994, over 4,400 evaluations have been conducted across more than 70 countries for both cultivation and food/feed use. To date, none have identified increased risk relative to conventional varieties. This article analyzes three decades of GM crop approvals, highlighting the consistency of scientific assessment processes. Fourteen crops underwent 692 cultivation and 1,891 food-release assessments for herbicide tolerance, while insect resistance accounted for 523 cultivation and 1,396 food-release assessments, largely involving *Bacillus thuringiensis* (Bt). The findings underscore that GM crops are equivalent in risk to conventional varieties, supporting reliance on established international scientific expertise, particularly in resource-constrained regions.

## 1. Introduction

Advancements in plant breeding technologies in the late 1980s and early 1990s led to the initial development and commercialization of genetically modified (GM) crops. Parallel to this were 1,183 scientific studies done in Canada, the European Union, and the United States that examined the potential for changes in risk from the commercialization of GM crop varieties, which led to the initial regulatory systems for products of agricultural biotechnology.^1^ The 1980s witnessed the standardization of risk assessment (RA) processes,^2,3^ utilized to assess whether there were new risks in any GM variety being assessed for commercialization. Risk assessments were based on comparing the risks of GM varieties to the risks of producing equivalent non-GM varieties. All crop production contains risks and by comparing the potential for changes in risk profiles, regulatory scientists could assess whether the new GM varieties differed in any way from the varieties being grown for food and feed consumption.

By 2025, 4,485 RAs had been conducted on GM crop varieties by scientific experts in 72 countries, 29 of which grew GM crops and the remainder importing them for food and feed purposes.^4^ These assessments resulted in approvals of 403 different traits across 29 different crop species. A 2015 study based on International Service for the Acquisition of Agri-biotech Applications (ISAAA) data found that of the 90 corn events approved, 26 were single trait varieties, either insect resistance (Bt varieties) or herbicide tolerance (HT varieties).^5^ Clearly, multiple RAs were conducted on the same variety by regulatory agencies in approving countries, without any variation in conclusions. Given the consistency in RAs, at what point is consensus reached whereby no further RAs are required as each additional RA does not provide additional knowledge or insight?

Countries that consistently battle food insecurity challenges stand to gain the most from innovative crop technologies, but can often be constrained by the fiscal and human resources required to conduct RAs and approve new crop varieties for commercial production. Risk assessments of some GM crop varieties have been conducted for 30 years without the identification of any changes in the risk of producing a GM crop from the risk of producing a non-GM crop. There is an opportunity to recognize the global scientific consensus that there has been no change in risk from the production of GM crop varieties, allowing food insecure countries to no longer spend scarce resources on repeating RAs that have not identified any changes over the past 30 years. To enable increased food production, food insecure countries need to be able to approve the commercialization of GM crops, if the GM crop variety has undergone a RA in another country and subsequently been approved for commercial production. Indeed, Hansen and Wingender^6^ identify that only one-third of the potential benefits from the adoption of GM crops have been realized due to regulatory barriers and bans.

Innovative technologies have benefited the production of GM crop varieties, especially through increased yields and reduced pesticide applications.^7,8^ With widespread and successful adoptions of GM crops, innovative solutions now need to be targeted at strategies that can reduce the time and cost of approving them for adoption in food insecure, resource constrained countries. This article reviews the RAs of GM crop varieties over the past 30 years, concluding that further RAs are an inefficient use of scarce time and fiscal resources for most GM traits and crop varieties.

## 2. Background

### 2.1. Risk Assessment

For approval, a GM crop must go under an RA that is country specific. In the RA, conditions like the GM plant’s capacity to cause harm to humans or other beneficial organisms (such as increased level of allergenics and/or anti-nutrients that are already part of the conventional organism consumed) exhibit altered interactions with plant pathogens, compete with and/or displace other plants, disseminate, and transfer genetic material to other organisms are all taken into consideration. This is necessary because the exogenous DNA introduced into GM food might come from an organism with no history of consumption.^9,10^

The GM RA can be divided into two components: contained use and non-contained use. Contained use refers to the initial development in the laboratory or greenhouse, as well as its storage, transportation, and disposal. In this case, the GM crop variety is not intended to be released into the environment, and appropriate containment measures must be implemented. Non-contained use refers to the deliberate release into the environment, which can include experimental release (field trials) conducted under specific risk management measures and/or commercial releases.^9^

Although there are similarities, the focus of the RA differs according to the intended use and level of experimental exposure. Even though contained use is not intended to be released in the environment, the evaluation of the risk for environmental and human health must be done, in case there is an unintended release, such as what happened with LL601 rice in 2006 in the USA. A biosafety containment level is also determined to minimize potential risks. Newly expressed proteins (NEPs) are a major focus in GM crop RAs, since their activity must be analyzed to be considered safe. Risk assessments for the same NEPs have been done several times, which accumulated large amounts of data that should be modernized based on the current knowledge on GM crops.^11^ Also, Brune et al.^12^ showed that some parts of the GM RA process for foods do not bring any relevant data, and therefore could be simplified. Koch et al.^13^ proposed a model for one global RA framework, eliminating redundancies between countries.

Data transportability (DT) has developed as a solution to eliminating redundancies between countries’ RAs. In 2013, Canada, Australia, and New Zealand agreed to collaborate to simplify the RA review process, considering the RA made in one country to all the others. In the proposed RA, Country A produces an RA on cultivation and use for the approved GM event. Country B wishes to import food, so it can access the RA of Country A and review some country-specific concerns that may not have been addressed yet. The same process applies to countries that want to cultivate and use the GM event.^13^ Japan adopted DT for GM corn in 2014 and GM cotton in 2019, while Argentina adopted DT for GM bean resistant to golden mosaic virus in 2019, developed by EMBRAPA in Brazil. In Africa, discussions have been held on adopting DT.^14^ Also in 2019, Paraguay implemented a simplified approval system that allows GM crops already assessed and authorized in trusted regulatory systems (based on Codex Alimentarius Guidelines for the Conduct of Food Safety Assessment of Foods Derived from Recombinant-DNA Plants) to undergo an expedited national review using foreign risk assessment data, while still considering local environmental conditions.^15^

The Codex Alimentarius Commission established the Ad Hoc Intergovernmental Task Forces on Foods Derived from Biotechnology with the objective of formulating general principles for the RA of foods produced through biotechnological processes and providing specific guidance for their safety evaluation. As a result of this initiative, several key documents were adopted by the Codex Alimentarius in 2003, including the Principles for the Risk Analysis of Foods Derived from Modern Biotechnology and the Guidelines for the Conduct of Food Safety Assessment of Foods Derived from Recombinant-DNA Plants and Microorganisms. In 2008 the same was adopted, but for animals. This task force was hosted in Japan for the last time in 2007 and is now dissolved.^10,16^ Each country has its own regulatory agencies that are responsible for the evaluation of the study, field trials, and release of new varieties, assessing the potential risks of the new variety to human and animal health, and the environment (Table 1).

**Table 1.** GM maize, cotton, soybean, canola major producers and their regulatory agencies.

Country	Top 5 producer of	GM approval of	Regulatory agencies	Reference
Argentina	Maize and soybean	Maize, cotton, and soybean	National Advisory Commission on Agricultural Biotechnology (CONABIA), National Service for Health and Agri-Food Quality (SENASA), and Technical Advisory Committee for the Use of GMOs (CTAUOGM).	CONABIA; SENASA; CTAUOGM; Vesprini et al. (2022)
Australia	Canola	Maize, cotton, soybean, and canola	Office of the Gene Technology Regulator and Food Standards Australia New Zealand (FSANZ), Therapeutic Goods Administration (TGA), Australian Industrial Chemicals Introduction Scheme (AICIS), Australian Pesticide and Veterinary Medicines Authority (APVMA), and Department of Agriculture, Fisheries and Forestry (DAFF).	Government of Australia; FSANZ; TGA; AICIS; APVMA; DAFF; Redden (2021)
Brazil	Maize, cotton, and soybean	Maize, cotton, and soybean	National Technical Commission on Biosafety (CTNBio); Internal Biosafety Committee (CIBio)	CTNBio; CIBio; Andrade et al. (2018)
Canada	Canola	Maize, cotton, soybean, and canola	Health Canada and Canadian Food Inspection Agency (CFIA)	Government of Canada; CFIA; Food Directorate; Lubieniechi, Gleim, and Smyth (2025)
China	Maize, cotton, soybean, and canola	Maize, cotton, soybean, and canola	Ministry of Agriculture and Rural Affairs (MARA)	MARA; Mu et al. (2025)
United States	Maize, cotton, and soybean	Maize, cotton, soybean, and canola	Food and Drug Administration (FDA), Environmental Protection Agency (EPA), and United States Department of Agriculture (USDA)	FDA; EPA; USDA; Roffman (2021)

Assessment by the World Health Organization^17^ confirms there are no effects shown on human health as a result of GM food consumption in the countries where they have been approved. The Government of Canada^10^ considers GM food as safe and nutritious as their non-GM counterparts. The US FDA – US Food and Drug Administration,^18^ the Argentine Council for Biotechnology Information and Development – ArgenBio,^19^ the Brazilian Ministry of Science, Technology and Innovation – MCTI,^20^ and Food Standards Australia New Zealand – FSANZ – Food Standards Australia New Zealand,^21^ have all confirmed GM crops and foods released on the market are safe for consumers.

### 2.2. World Panorama on GM Crops

The first modified organism was in 1973, a bacterium that is still used, Escherichia coli. It took more than 10 years for the technology to be applied to plants, and the first one to be modified was tobacco, resistant to tobacco mosaic virus, commercialized in China. In 1992, the first GM tomato was approved for cultivation in the USA, and for human consumption in 1994.^1,22^ Also in 1994, GM canola was approved in Canada. In 1995, cotton, maize, potato, and squash were approved, followed by soybean in 1996, chicory and papaya in 1997, flax, sugar beet, and sweet pepper in 1998, melon in 1999, and rice in 2000. The latest crop to be approved was cowpea in 2019 (Table 2).

**Table 2.** First GM events released for food use (direct use or additive) on the market for each crop.

Crop	Year	Country	GM trait	Event name
Canola	1994	CAN	Herbicide tolerance	GT73
			Herbicide tolerance, pollination control system	RF1
Tomato		USA	Delayed fruit softening, antibiotic resistance	FLAVR SAVR
Cotton	1995	USA	Herbicide tolerance, antibiotic resistance	BXN10211
				BXN10215
				BXN10222
				MON1445
				MON1698
			Insect resistance, antibiotic resistance	MON1076
				MON531
				MON757
Maize		USA	Herbicide tolerance, insect resistance, antibiotic resistance	BT176
			Herbicide tolerance, antibiotic resistance	T14
				T25
Potato		CAN USA	Insect resistance, antibiotic resistance	BT06
				BT10
				BT12
				BT16
				BT17
				BT18
				BT23
		CAN		SPBT02-5
				SPBT02-7
Squash		USA	Viral disease resistance	ZW20
Soybean	1996	ARG CAN MEX CHE URY	Herbicide tolerance	GTS 40–3-2
Chicory	1997	USA	Herbicide tolerance, pollination control system, antibiotic resistance	RM3-3
				RM3-4
				RM3-6
Papaya		USA	Viral disease resistance, antibiotic resistance, visual marker	55–1
Flax	1998	CAN USA	Herbicide tolerance, antibiotic resistance, product quality	FP967
Sugar beet		USA	Herbicide tolerance, antibiotic resistance	T120-7
			Herbicide tolerance, visual marker	GTSB77
Sweet pepper		CHN	Viral disease resistance	PK-SP01
Melon	1999	USA	Delayed ripening, antibiotic resistance	Melon A
				Melon B
Rice	2000	USA	Herbicide tolerance	LLRICE06
				LLRICE62
Alfalfa	2004	USA	Herbicide tolerance	J163
				J101
Wheat		AUS COL NZL USA	Herbicide tolerance	MON71800
Plum	2009	USA	Viral disease resistance, antibiotic resistance, visual marker	C-5
Bean	2011	BRA	Viral disease resistance	EMBRAPA 5.1
Sugarcane		IDN	Drought stress tolerance, antibiotic resistance	NXI-1T
Eggplant	2013	BGD	Insect resistance, antibiotic resistance	EE1
Apple	2015	CAN USA	Antibiotic resistance, non-browning	GS784
				GD743
Eucalyptus		BRA	Antibiotic resistance, volumetric wood increase	H421
Pineapple	2016	USA	Delayed ripening, modified fruit color	EF2-114
Safflower	2017	ARG	Stacked herbicide tolerance	IND-10003–4 ×IND-10015–7
Cowpea	2019	NGA	Insect resistance	AAT709A

Source: Information gathered from ISAAA.^23^ AUS: Australia, ARG: Argentina, BGD: Bangladesh, BRA: Brazil, CAN: Canada, CHE: Switzerland, CHN: China, COL: Colombia, IDN: Indonesia, MEX: Mexico, NGA: Nigeria, NZA: New Zealand, URY: Uruguay, and USA: United States.

In 1973, the safety of GM organisms started being discussed, and two years later, at the Asilomar Conference, the first guidelines on laboratory practices to handle those organisms were proposed and adopted, which later formed the basis for regulatory frameworks. The Organization for Economic Cooperation and Development (OECD) published their first report on GM biosafety at the same time that the first field trial of GM crops started in 1986. In the same year, a Coordinated Framework for the Regulation of Biotechnology was established by the USA. In 1993, the OECD published three papers on the safety of biotechnology for food crops, filling gaps and targeting uncertainties regarding the subject.^1,24,25^ In 1995, a discussion regarding the risk assessment of GM products was brought together by the OECD Working Party on the Harmonization of Regulatory Oversight in Biotechnology (WP-HROB), including national authorities from OECD countries and partners to ensure that the scientific methodologies underpinning RAs were harmonized.^26^ This increases the opportunity for acceptance of information from other countries. Nevertheless, this is not worldwide acceptance, and repeated RAs increase commercialization costs for new adoptions of the same variety. The cost of regulation for a single new GM trait is estimated by AgBioInvestor^27^ at US$43.2 million (2017–2022), which is greater than it was in 2008–2012 period (US$35.1 million). The value of the 2017–2022 period represents 37.6% of the total costs of the development of a GM trait.

Since the main challenges for farmers are to control weed and insects, the main GM traits introduced are herbicide tolerance and insect resistance.^28,29^ According to Noack et al.^30^ GM soybeans and canola have herbicide tolerance as their predominant trait, while GM cotton and maize have insect resistance as their predominant trait. Some varieties present both as stacked traits.

### 2.3. Herbicide Tolerance

No definition of a weed is shared by all scientists, but the understanding is the same: a plant growing where it is not desired.^31^ Weeds have been a huge problem in agriculture, because of their capacity to reproduce quickly, competing for nutrient, water, and sunlight use with crops, reducing the yield of the crops significantly, as well as affecting the development and growth of crops.^32^ Weeds can also harbor diseases and insects, reduce crop quality, and clog irrigation systems.^33^ Therefore, farmers need to control weeds in their fields, and they do so by using tillage, hand weeding, and/or herbicide use. Tillage damages soil health and leads to more greenhouse (GHG) emissions, so farmers started to avoid this technique.^34^

GM herbicide tolerant crops have been approved for 30 years in 63 countries and do not pose environmental and/or health risks as compared to their non-GM counterparts. The main targets in herbicide tolerance are glyphosate and glufosinate, since those are broad-spectrum herbicides which will control most green plants. Also, these herbicides are not persistent and have a minimal direct impact on animal life.^35,36^

### 2.4. Insect Resistance

The insect resistance trait introduced in maize, cotton, and soybean is usually from a bacterial donor, especially from *Bacillus thuringiensis* (Bt). This bacterium naturally has different delta endotoxins that damage the midgut lining (insect digestive system) of the insect (either lepidopteran or coleopteran). Those different types of endotoxins can be introduced in the plant, that will start producing the endotoxin that damages the insects, conferring resistance. There are other bacteria species that work as gene donors for crops. Bt crops have been largely adopted around the world for being highly competitive from an agronomic perspective. One of the major benefits of insect resistance crops is the reduction on the use of insecticides.^37–39^ However, insects can develop resistance over time, so there are insect resistance management plans for each target insect.^40,41^

## 3. Methods

The data presented in this article was taken from ISAAA and FAOSTAT websites. The ISAAA data was collected in August 2025, for a period from 1994 to 2025, and for 2023 for FAOSTAT, which is the latest released data. The number of risk assessments was estimated as follows:

$$RA_{C}=\underset{e}{\sum }A_{e}$$

where RA is the total number of risk assessments for crop *c*, *e* is the GM event, and A_e_ is the number of countries that have approved event *e* for food or cultivation. The European Union countries was considered as one A_e_, and so was Australia and New Zealand, since they are under the same regulatory agency and account for only one risk assessment. After 2017, food/cultivation release of GM events in Australia, New Zealand, and Canada was considered as one risk assessment because of the Safety Assessment Sharing Initiative between Health Canada and Food Standards Australia New Zealand (FSANZ).^42^ GM events released after 2020 in Bangladesh, Bhutan, India, and Sri Lanka were also considered as one risk assessment because of harmonization initiative in South Asia.^42^ After 2022, Argentina and Brazil started a Memorandum of Understanding for Cooperation in Biosafety of Modern Biotechnology Products, but this cooperation is not necessarily applied to all GM events, so each country was considered as one risk assessment.^42^

The doughnut charts and the 100% stacked bar chart were both developed using Excel, and the vertical bars chart combined with line plus symbol for country and GM events approval was developed in SciDavis. The doughnut chart of the GM traits for each crop was considered above 1%, anything below was gathered in “others” section. The maps were created in Excel.

## 4. Results

**Figure 1.**
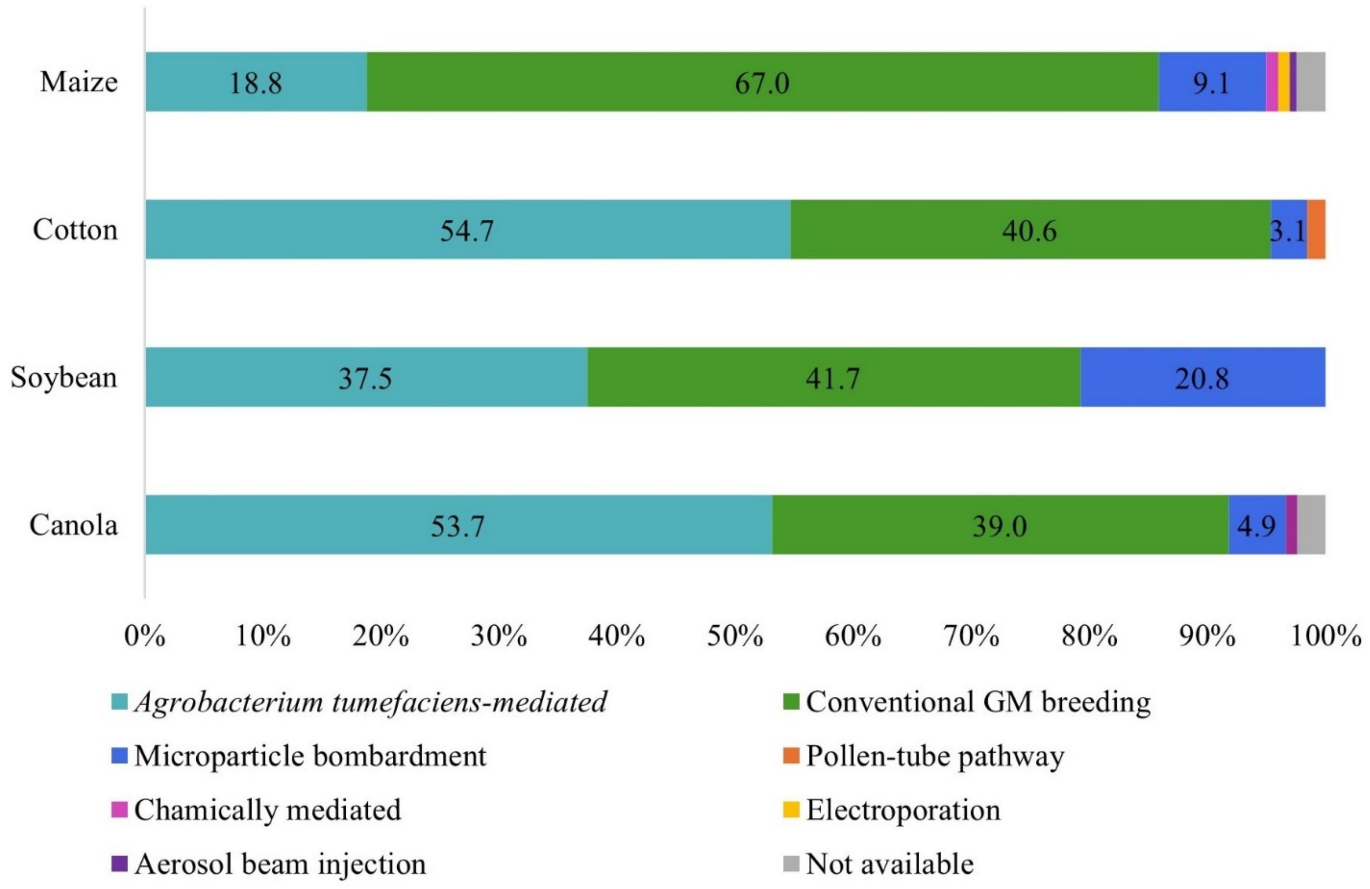
Method of trait introduction on GM maize, cotton, soybean, and canola approved for food. Source: ISAAA^23^.

From 645 total GM events approved, 469 of them were approved for food in 65 countries, including 24 different crops, and 443 were approved for cultivation in 64 countries, including 30 different crops. Of those, the main crops were maize, cotton, soybean, and canola. A GM trait can be directly introduced in the crop by different means, with Agrobacterium tumefaciens-mediated plant transformation the most common among the top four crops (Figure 1), followed by microparticle bombardment. One of the major ways to develop GM crops is by breeding GM crops with different desirable traits. Although conventional breeding is not considered a GM conventional method, it was used on GM parentals, and the resultant offspring still had to go through the same RA as the methods of direct trait introduction.

**Figure 2.**
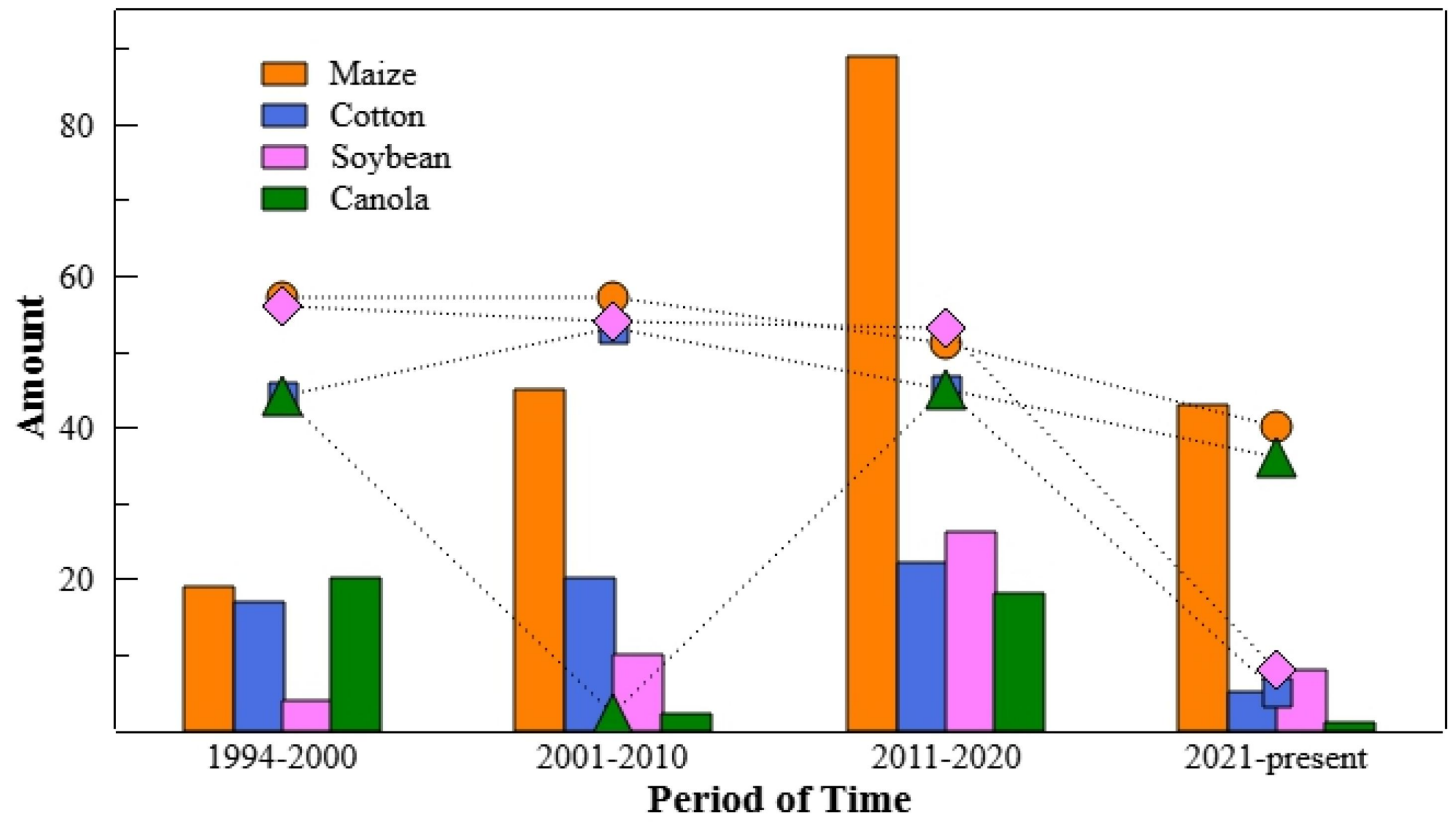
Number of GM event approvals (bars) for maize, cotton, soybean, and canola; and number of countries that approved those events (dotted line + symbol). Source: ISAAA^23^.

Maize had the highest number of GM events approved between 2011 and 2020, with 88 events approved for food. Cotton and soybean also had a higher number of approvals between 2011 and 2020, with 22 and 25 approvals for food in the period, respectively. In the 1994–2000s interval, canola had the greatest amount of GM events approved, which drastically reduced between 2001 and 2010, and grew again in the 2011–2020 period. Over the last five years, canola had one GM event approved in 36 countries, with herbicide tolerance trait (Figure 2).

There were 88 institutions that developed a GM event either for cultivation, food, and/or feed. However, among the top four crops, only cotton had public institutions (Chinese Academy of Agricultural Sciences, Central Institute for Cotton Research and University of Agricultural Sciences Dharwad – India, and Texas A&M AgriLife Research University) that developed GM events, which accounts for less than 5% of the total institutions that developed GM events for cotton.

### 4.1. GM Maize

**Figure 3.**
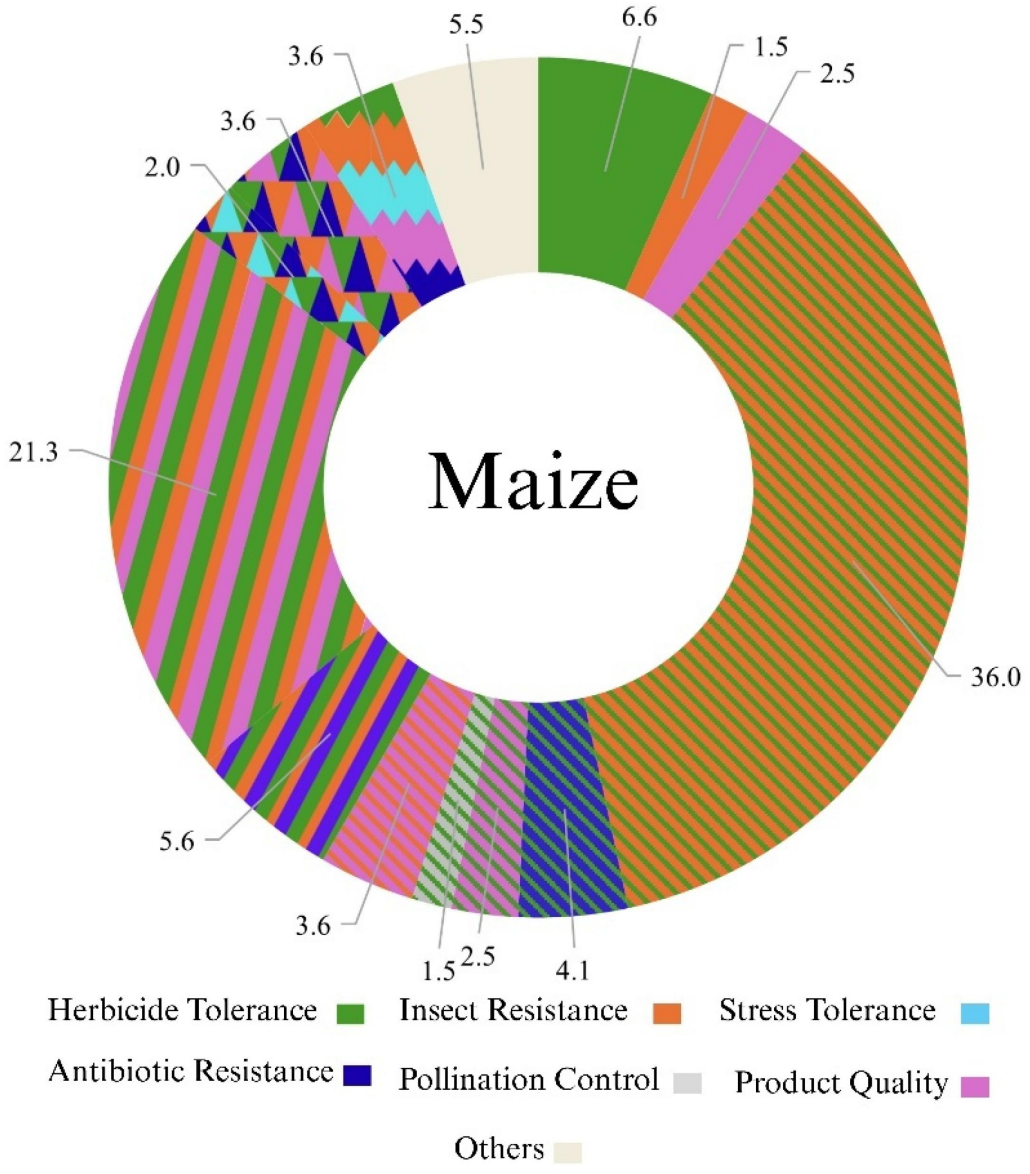
Stacked and singular GM maize traits approved for food. Data present on “others”: 0.5% stacked insect resistance and antibiotic resistance, 0.5% stacked insect resistance and abiotic stress tolerance, 0.5% stacked abiotic stress tolerance and antibiotic resistance, 0.5% stacked pollination control system and visual marker, 0.5 stacked herbicide tolerance, insect resistance, and abiotic stress tolerance, 0.5% stacked herbicide tolerance, abiotic stress tolerance, and antibiotic resistance, 0.5% stacked herbicide tolerance, abiotic stress tolerance, and product quality, 0.5% stacked herbicide tolerance, abiotic stress tolerance, antibiotic resistance, and product quality, 0.5% stacked insect resistance, abiotic stress tolerance, antibiotic resistance, and product quality, and 1% stacked herbicide tolerance, pollination control system, and antibiotic resistance. Source: ISAAA^23^.

**Figure 4.**
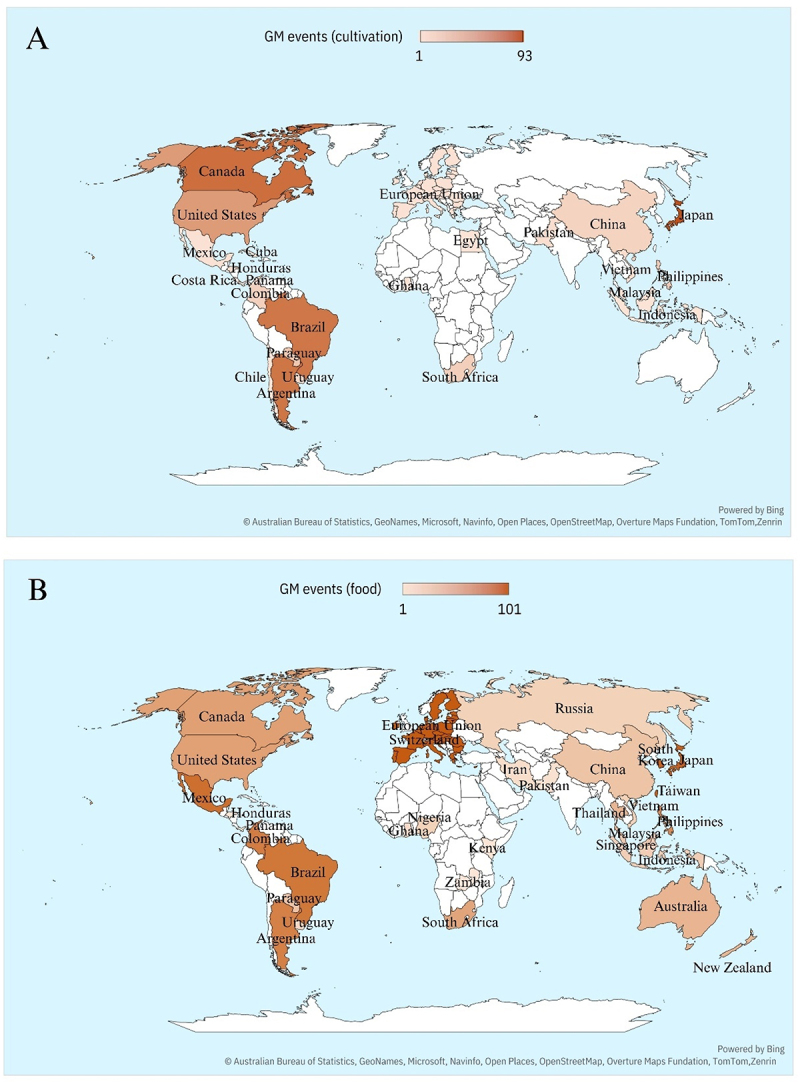
Maize GM events approved around the world for cultivation (A) and for food (B). Source: ISAAA^23^.

Of the 197 GM maize events approved for food, only 6.6% were solely for herbicide tolerance, 2.5% for modified product quality, 1.5% for insect resistance, and the remaining 89.3% were stacked traits (Figure 3). GM maize was approved for food in 58 countries (Figure 4). With major producers being the USA, China, Brazil, Argentina, and India (non-GM maize).^43^ From those, only India had not approved GM maize events for cultivation and food. The top five exporters were Brazil, USA, Ukraine, Argentina, and Romania, India was in 12th position. The top five importers were China, Mexico, Japan, Vietnam, and Republic of Korea.^23,43,44^ Countries that adopted at least one GM maize event represent 83% of all maize production worldwide and countries that approved at least one GM maize event for food account for 76.5%.

### 4.2. GM Cotton

**Figure 5.**
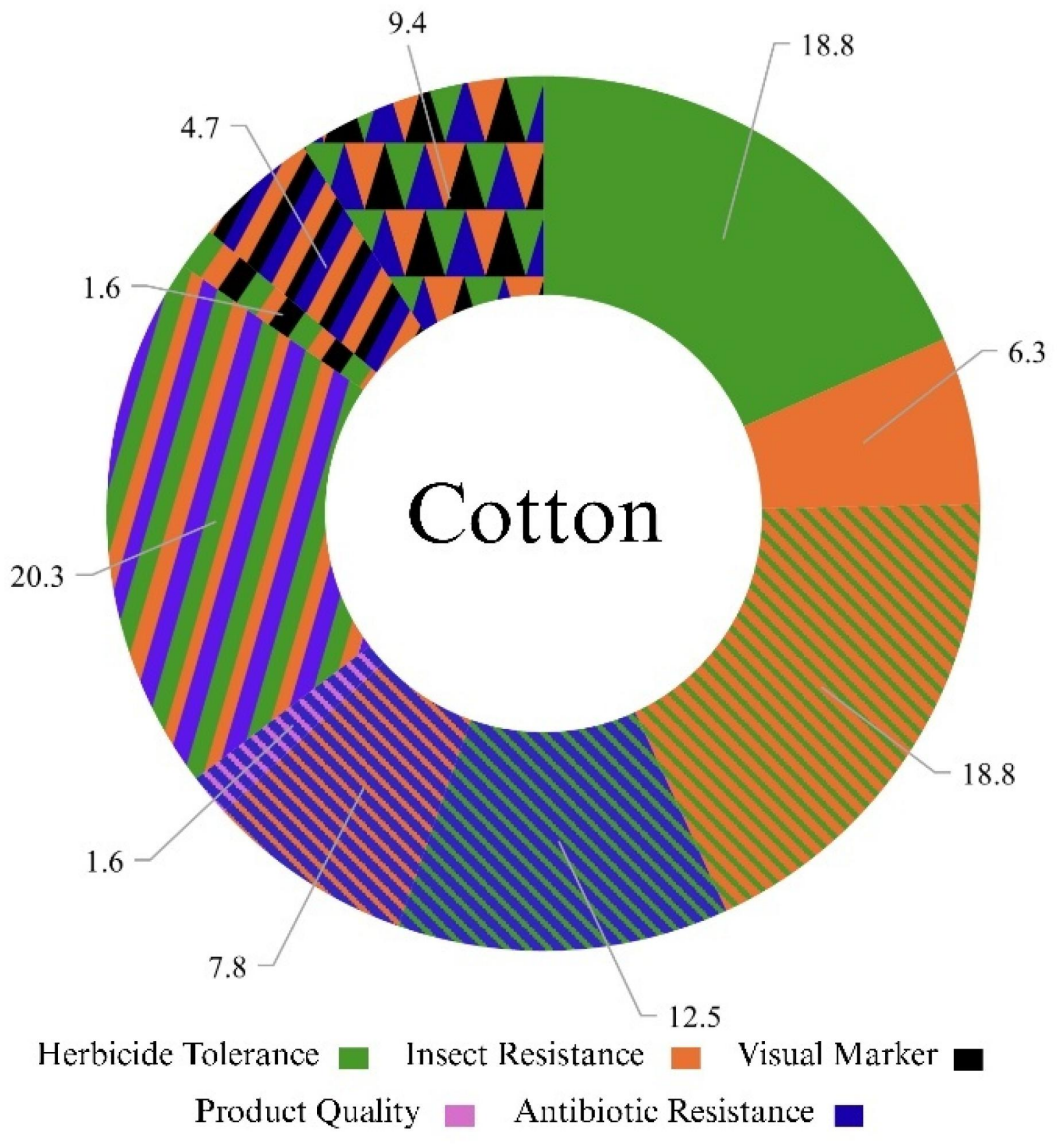
Singular and stacked traits of GM cotton approved for food. Source: ISAAA^23^.

**Figure 6.**
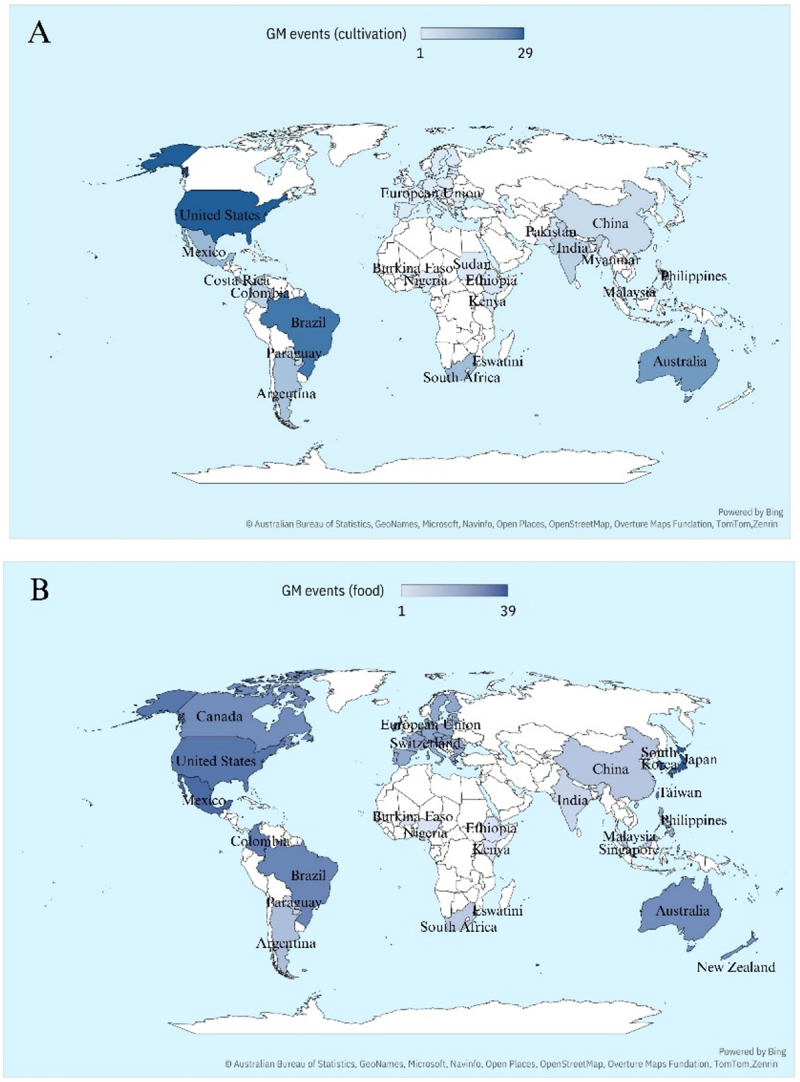
Cotton GM events approved around the world for cultivation (A) and for food (B). Source: ISAAA^23^.

Cotton has 64 GM events approved for food with 18.8% of events developed for herbicide tolerance trait only, 6.3% were developed for insect resistance, and 74.9% were stacked traits (Figure 5). GM cotton was approved for food in 51 countries. The countries that have adopted at least one GM cotton event represented 83% of all cotton production worldwide. The top five producers of cotton have all adopted GM cotton: China, India, USA, Brazil, and Pakistan. From the top five exporters, only Turkey did not approve GM cotton events for cultivation and food. The other top exporters were India, Brazil, South Africa, and USA. From the top five importers, only China and USA approved GM cotton events for cultivation. From the others, only Japan approved GM cotton events for food. The other two countries (Iran and Tajikistan) did not have any GM cotton events approved (Figure 6).^23,43,44^

### 4.3. GM Soybean

**Figure 7.**
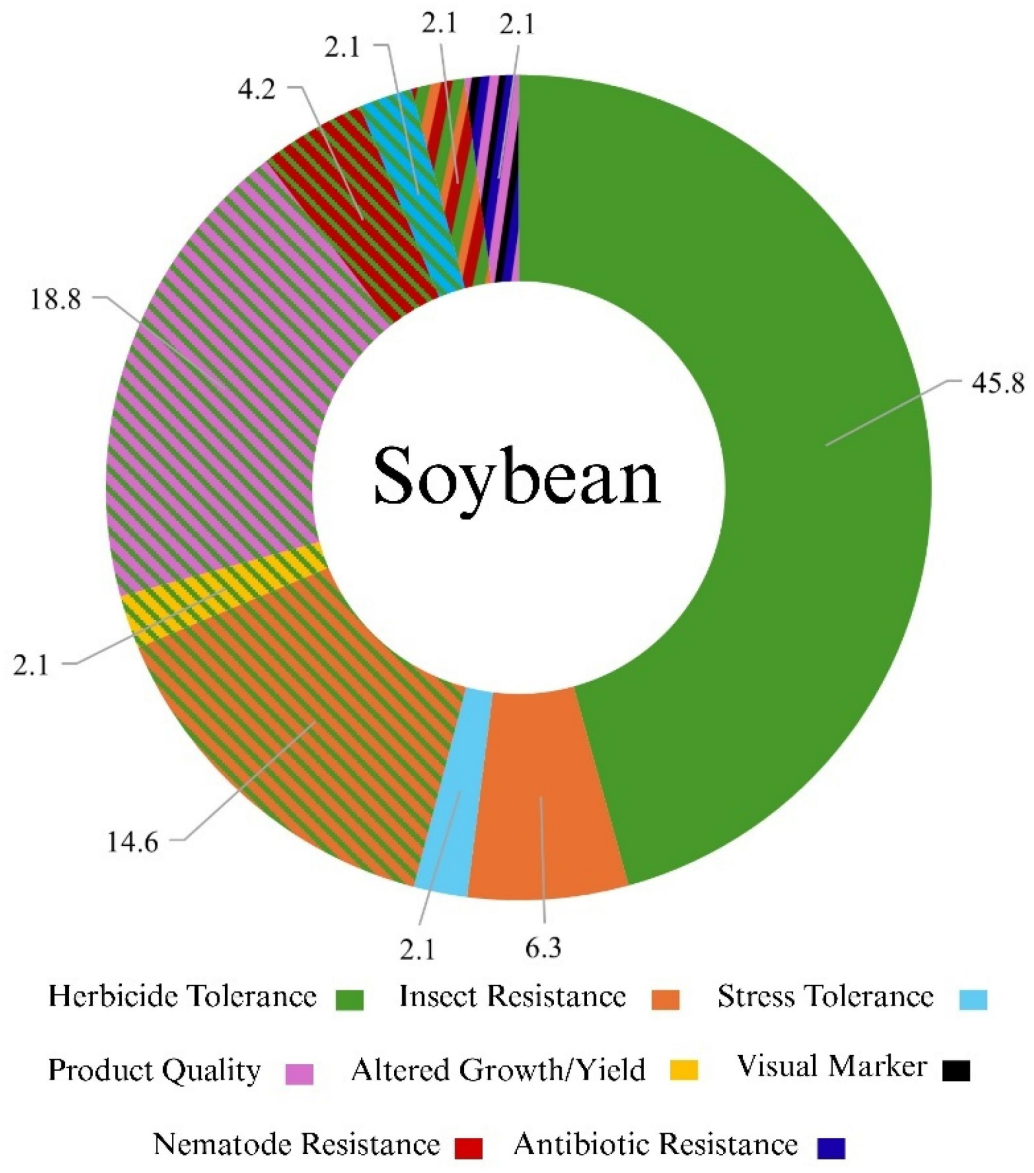
Stacked and singular GM soybean traits approved for food. Source: ISAAA^23^.

**Figure 8.**
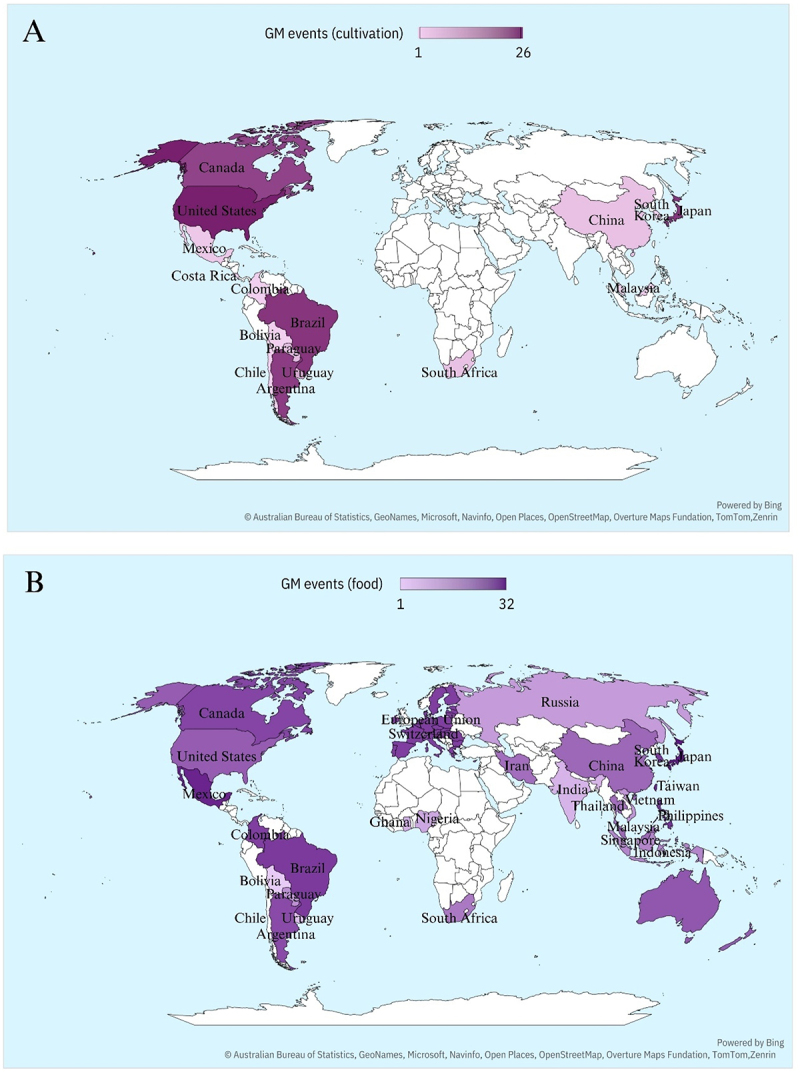
Soybean GM events approved around the world for cultivation (A) and for food (B). Source: ISAAA^23^.

There were 48 GM soybean events approved for food with 45.8% of those for herbicide tolerance alone, 6.3% for insect resistance, and 2.1% for abiotic stress tolerance. The remaining 45.8% were stacked traits^23^ (Figure 7). GM soybean was approved for food in 55 countries (Figure 8). The countries that adopted at least one GM soybean event represented more than 90% of all soybean production worldwide. The top five producers of soybean were Brazil, USA, Argentina, China, and India. From those, only India has approved GM soybean event only for food, the others approved it for both food and cultivation. From the top five main exporters, Brazil, USA, Paraguay, Canada, and Ukraine, only the last country did not approve GM soybean events for cultivation or food. The top five importers of soybeans all have approved GM soybean events for food including China, Argentina, Mexico, Netherlands, and Spain.^23,43,44^

### 4.4. GM Canola

**Figure 9.**
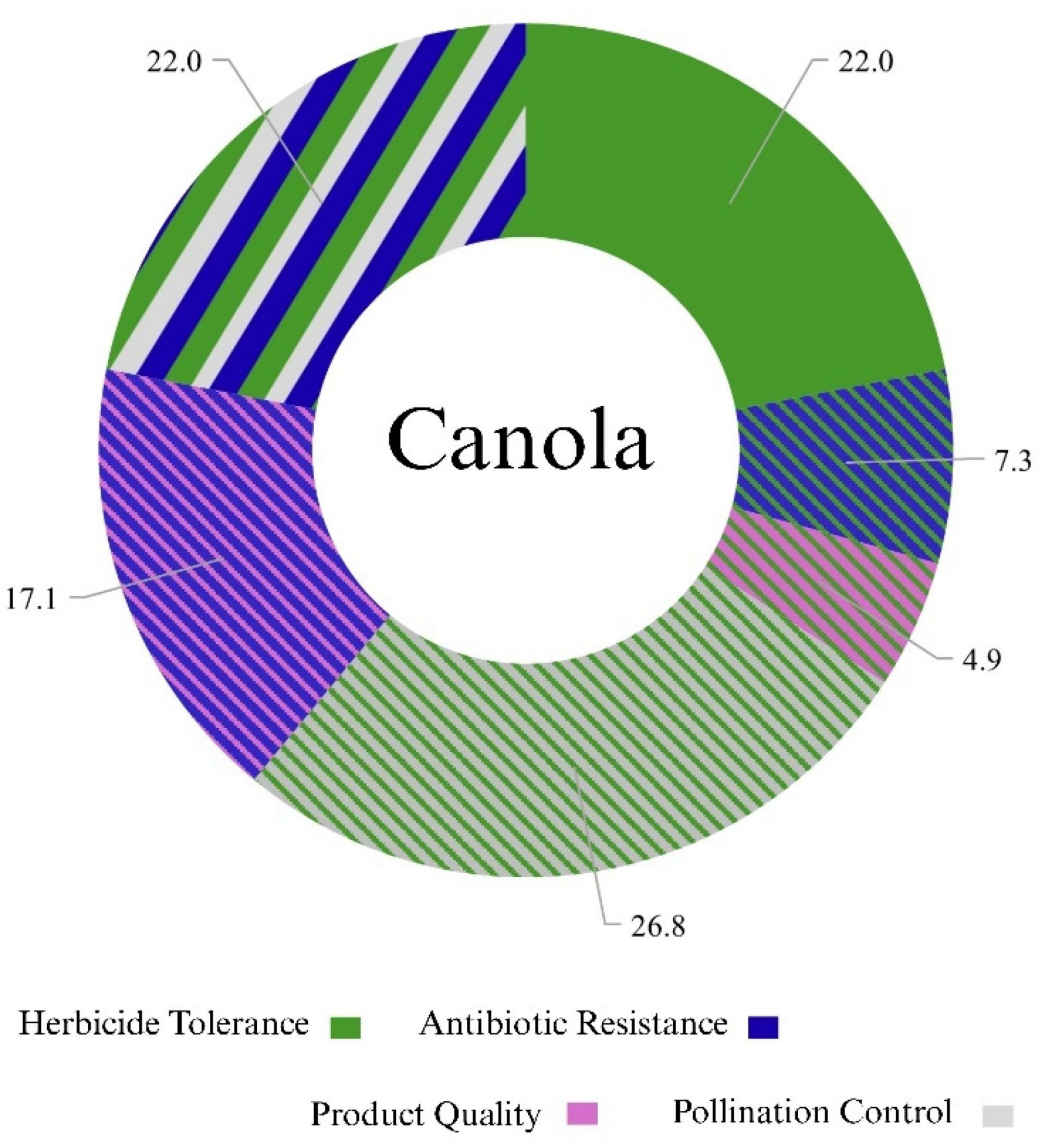
Stacked and singular GM canola traits approved for food. Source: ISAAA^23^.

**Figure 10.**
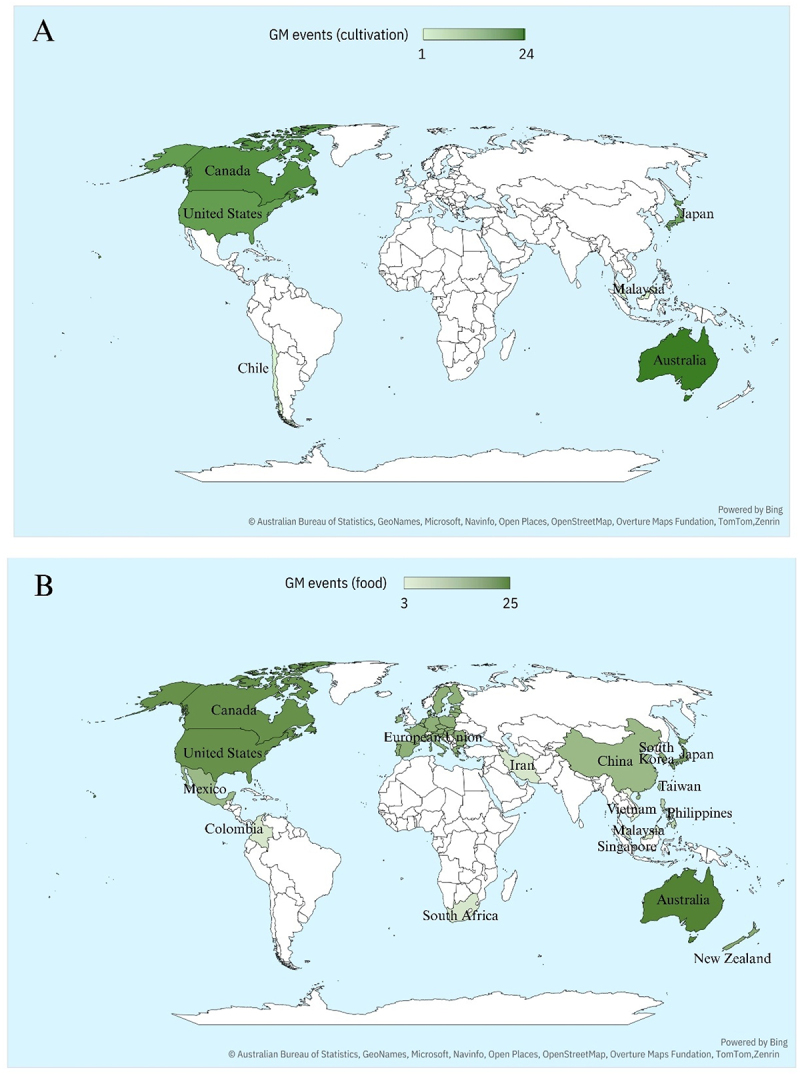
Canola GM events approved around the world for cultivation (A) and for food (B). Source: ISAAA^23^.

Canola had 41 GM events approved for food with herbicide tolerance (17.1%), and stacked traits (82.9%) events approved (Figure 9). GM canola has been approved for food in 44 countries (Figure 10). The countries that adopted at least one GM canola event represent 31.8% of all canola production worldwide. Of the top five canola producing countries Canada, China, India, Australia and France, only two of those approved GM canola events for cultivation (Canada and Australia), although for food this number is higher. Only India did not approve GM events for either cultivation or food. The main exporters of canola were Canada, Australia, Ukraine, Romania, and Belgium, and only Ukraine had no GM approvals. The top main importers all have approved GM canola for food, being them Germany, China, Belgium, Japan, and France.^23,43,44^

**Figure 11.**
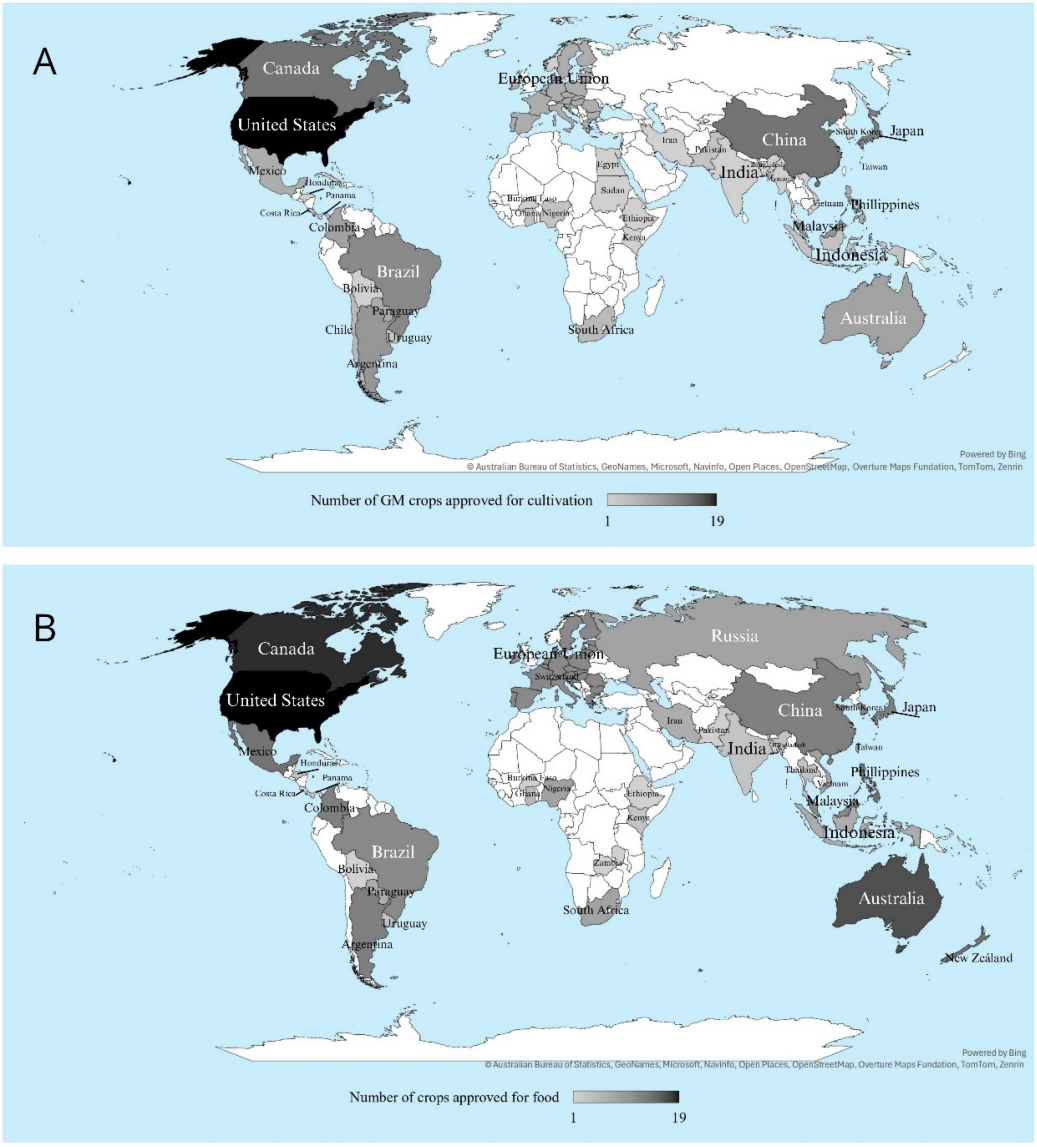
Countries that adopted GM crops for cultivation (A) and for food (B). Source: ISAAA^23^.

The countries that approved GM crop events for both cultivation and food are Argentina, Australia, Bangladesh, Bolivia, Brazil, Burkina Faso, Canada, China, Colombia, Costa Rica, Cuba, Egypt, Eswatini, Ethiopia, Ghana, Honduras, India, Indonesia, Iran, Japan, Kenya, Malaysia, Mexico, Myanmar, Nigeria, Norway, Pakistan, Panama, Paraguay, Philippines, South Africa, South Korea, United States, Uruguay, Vietnam, and the European Union. Whilst, Chile, Egypt, Cuba, and Sudan have approved GM crop events exclusively for cultivation and New Zealand, Taiwan, Thailand, and Switzerland approved GM crop events just for food (Figure 11).

### 4.5. Herbicide Tolerance Trait

To confer tolerance to herbicides, most genes inserted into crops came from bacteria. Bacteria like *Bacillus licheniformis* have an enzyme that catalyzes the inactivation of glyphosate, which was used in corn, soybean, and canola to confer resistance to this herbicide. Other species like *Ochorobactrum anthropi* and *Agrobacterium tumefaciens* had similar effects, with enzymes that confer resistance to glyphosate in corn, soybean, canola, and cotton. For glufosinate herbicide tolerance, a gene derived from bacteria *Streptomyces viridochromogenes* was introduced in maize, soybean, canola, and cotton. *Streptomyces hygroscopicus* were used for either glyphosate and glufosinate herbicide tolerance in canola and just for glufosinate in corn and cotton. Dicamba has tolerant corn, soybean, canola, and cotton events, with *Stenotrophomonas maltophilia* bacteria as the gene donor. Oxynil herbicide tolerance was induced in canola and cotton by an enzyme from bacteria *Klebsiella pneumoniae*. *Delftia acidovorans* bacteria acted as a gene donor to cotton and soybean to confer tolerance to 2,4-D herbicide, also *Sphingobium herbicidovorans* was used as gene donor for corn and soybean tolerance to 2,4-D herbicide, and corn tolerance to FOPs (aryloxyphenoxypropionate) herbicides. *Pseudomonas fluorescens*’ gene was introduced on cotton and soybean for isoxaflutole herbicide tolerance, and tolerance to HPPD (p-hydroxyphenylpyruvate dioxygenase) inhibiting herbicides in soybean. Another species of Streptomyces genera, *Streptomyces sviceus* was used as gene donor to confer tolerance to glyphosate in corn. *Anthrobacter globiformis* bacteria was also used to confer tolerance to glyphosate in corn.

The *Arabdopsis thaliana* plant was used as gene donor to confer resistance to herbicide imazamox in canola, and imidazolinone in soybean. For GM cotton tolerant to herbicide, a plant gene from *Nicotiana tabacum* was used. Corn (*Zea mays*) was used as a gene donor to cotton and soybean to confer tolerance to glyphosate; and to another varieties of corn to confer tolerance to herbicides such as glyphosate, sulfonylurea, and imidazolinone. Soybean naturally resistant to sulfonylurea herbicide was used as gene donor to a nonresistant soybean variety. For mesotrione herbicide tolerance in soybean, rice (*Oryza sativa*) and oat (*Avena sativa*) have been used as gene donors.

### 4.6. Insect Resistant Trait

For maize, bacterial genes were used to confer resistance to insects. *Bacillus thuringiensis* was the most common bacteria used to confer resistance to lepidopteran and coleopteran, especially corn rootworm. There were also other bacteria species that played a role in insect resistance in corn, like *Solanum tubrosum*, conferring resistance to multiple insects; *Pseudomonas chlororaphis* was used to confer resistance to coleopteran; *Ophiglossum pendulum* was also used to confer resistance to coleopteran, specifically corn rootworm, likewise *Brevibacillus laterosporus*. Another way of conferring this resistance in corn was using a double-stranded RNA transcript containing part of a target gene of the insect itself (*Diabrotica virgifera virgifera* - corn rootworm) resulting in the downregulation of the targeted gene, leading to the insect’s death.

For cotton, bacterial genes were used to confer resistance to insects. *Bacillus thuringiensis* was the most common bacteria used to confer resistance to lepidopteran, but it has also been used to confer resistance to hemipteran. Another bacterial species, *Vigna ungiculata* has been used to confer resistance to a wide range of insect pests. For soybeans, *Bacillus thuringiensis* was solely responsible for conferring insect resistance, exclusively for lepidopteran. Canola did not have any events with insect resistance trait approved.

### 4.7. Risk Assessments

There was a decrease in the number of risk assessments calculated after consideration of the agreements in place. For herbicide tolerance trait, there was a 4% decrease in the number of RAs for food release with the agreements, and 3.5% RAs decrease for insect resistance trait, also for food release. The crops most affected by the agreements were potato and bentgrass for herbicide tolerance trait (9%), and potato for insect resistance trait (10%). Potato was the crop with the most reduction in the number of RAs because of the agreements already in place.

There were 440 GM events approved with herbicide tolerance trait on alfalfa, canola, carnation, chicory, cotton, bentgrass, eucalyptus, flax, maize, polish canola, potato, rice, soybean, sugar beet, sugarcane, tobacco, and wheat. From those events, 1,896 RAs were made for food release, and 695 for cultivation.^23^ This includes 997 RAs for maize and 344 RAs for soybean, both for food (Table 3).

**Table 3.** Risk assessments (RAs) made for herbicide tolerance and insect resistance traits in GM crops, released for food and/or cultivation.

	RAs for Herbicide tolerance			RAs for Insect resistance		
	Food	Cultivation	Events	Food	Cultivation	Events
Alfalfa	26	12	4	0	0	0
Argentine Canola	192	74	37	0	0	0
Carnation	0	14	4	0	0	0
Chicory	3	3	3	0	0	0
Cotton	278	93	52	247	99	53
Cowpea	0	0	0	2	2	1
Creeping Bentgrass	0	1	1	0	0	0
Eggplant	0	0	0	2	2	1
Eucalyptus	7	7	7	1	1	1
Flax	2	2	1	0	0	0
Maize	997	374	267	932	346	260
Polish Canola	0	4	4	0	0	0
Poplar	0	0	0	0	2	2
Potato	10	2	4	98	38	30
Rice	11	3	3	3	3	3
Soybean	344	97	46	101	25	11
Sugar Beet	22	7	4	0	0	0
Sugarcane	1	0	1	12	5	8
Tobacco	0	0	1	0	0	0
Tomato	0	0	0	2	1	1
Wheat	3	0	1	0	0	0
Total	1,896	693	440	1,400	524	371

There were 372 GM events with insect resistance trait, including cotton, cowpea, eggplant, eucalyptus, maize, poplar, potato, rice, soybean, sugarcane, and tomato. 1,398 RAs have been made for food release, and 520 for cultivation (Tables 1 and 3). Of all those RAs, approximately 97% were for GM events released for food using *B. thuringiensis* as gene donor.^23^ For maize alone there are 932 RAs using Bt as gene donor for food, and 101 RAs using Bt as gene donor for food in soybean.

## 5. Discussion

Conventional breeding usually refers to the process humans have been doing for thousands of years involving artificial selection. When it comes to GM plants, conventional breeding is the crossing between two or more GM parentals. If the GM parentals have already been risk assessed and proven as safe as their non-GM counterpart, there is no safety gained by repeating the RA for the offspring, since conventional breeding of non-GM parentals is not regulated. Of all GM maize events developed, 67% were through GM conventional breeding, which is higher than the percentage of GM maize developed by methods of direct trait introduction like *Agrobacterium tumefaciens*-mediated transformation (18.8%) and microparticle bombardment (9.1%) (Figure 1). This increases the costs of GM maize regulation by approximately US$5.6 billion, with no additional benefit for consumers and the environment.^23,27^

Public institutions have a large role in the research and development of new crop varieties in all countries, but not as much on the development of GM crop varieties because of the limited funding to go through the regulation process. If there were more public investments on GM events approval, there could be more benefits to society, such as with Golden Rice.^45,46^

Being the world’s largest used crop, maize is also the largest GM crop cultivated. There are more approvals for food release than for cultivation, because the number of countries that plant GM crops are smaller than the number of countries that import them for food. The EU is a great example, having only two GM maize approvals for cultivation, but 101 approvals for food. The EU has only 11 GM events approved for cultivation, divided into carnation, cotton, maize, and potato crops. However, for food, this number rises to 161 GM events divided into canola, cotton, maize, potato, soybean, and sugar beet.

The larger GM producing countries like Argentina, Brazil, Canada, and the USA had little difference between the number of approvals for cultivation and for food. Argentina had 103 GM events approved for cultivation, against 114 for food; Brazil had 130 GM events approved for cultivation and 150 for food; Canada had 153 GM events approved for cultivation and 171 GM events approved for food; and the USA had 190 GM events approved for cultivation, and 202 for food. In general, there are more approvals for food (469 GM events) than for cultivation (443 GM events). More countries approve GM events for food than for cultivation is true for all four major GM crops.

Maize is one of the world’s leading staple cereals,^47^ which together with rice and wheat comprises 42% of the world’s food calories.^48^ Maize has a versatile use, such as for food, livestock feed, industrial, and energy production. In sub-Saharan Africa, Latin America, and few countries in Asia, it contributes over 20% of food calories. Therefore, maize plays a pivotal role in food security and production worldwide. Because of the growing demand, concerns have been raised on how to improve productivity while mitigating climate change.^47^ Genetically modifying maize is one of the ways to improve yield, while using less land and agricultural inputs.

Cotton is the world’s most important natural fiber crop, being 35% of annual fiber demands.^49^ Besides being used as fiber, cotton can also be used as protein source for some animals, and to make oil from the cottonseed, that is used for human consumption.^50^ Soybean is another multi-purpose crop. It is one of the most important oilseed and biodiesel, besides being a protein source for human food and animal feed.^51,52^ Canola is the world’s second largest oilseed crop,^53^ with Canada the largest canola producer worldwide.

The same organisms can be used as gene donors to different crops to confer tolerance to herbicide and resistance to insects. As an example of insect resistance trait, maize had 928 RAs for 165 different events using the same bacteria as gene donor. The first GM insect-resistant events approved for food using *B. thuringiensis* were cotton, maize, and potato in 1995, consolidating it as a safe gene donor for 30 years. One year before the release of insect-resistant crops for food using *B. thuringiensis*, the first GM event on herbicide tolerance was released for food in canola. The gene donors in this case were both *A. tumefaciens* and *O. anthropic* for glyphosate tolerance. Both bacteria were later used to confer herbicide tolerance in cotton, maize, and soybean. Herbicide tolerance and insect resistance are two well-established traits on GM events for a diverse range of crops that had more than a thousand RAs (Table 3).

Some countries are more aligned with their risk assessments and regulations. OECD has a system called Mutual Acceptance of Data (MAD), with 40 countries having a multilateral agreement where chemical testing is conducted using OECD standard test methods and in accordance with OECD Principles of Good Laboratory Practices (GLP). Besides the OECD member countries participating in MAD System, other nonmembers of OECD also joined, including Argentina, Brazil, India, Malaysia, Singapore, South Africa, and Thailand. This allows the participant countries to accept data generated in other jurisdictions for regulatory decision-making, saving time and resources while relying on the use of standardized test guidelines and GLP compliance.

A similar concept based on increased data transportability could be considered for GM risk assessments, so that money, time, and resources are not unnecessarily spent on repeating already established studies for GM varieties. Such shared data could be used to facilitate and streamline the risk assessment process across countries, while still allowing national authorities to consider country-specific environmental conditions when needed.^54,55^

### 5.1. Impacts on the Environment and Crop Yield

GM yield gains reduce land use, therefore deforestation and all the greenhouse gas (GHG) emissions that come with it. By reducing tillage, GM crops can reduce GHG emissions and increase carbon sequestration.^56^ With the potential for higher yield in GM crops, there is a reduction in agricultural GHG emissions from land use, that would account for 20% of all agricultural GHG emissions.^57^ Additionally, GM crops result in up to 37% less pesticide use.^7^ One way of increasing effective yield is adopting GM crops with insect resistance and herbicide tolerance traits, as they help reduce crop damages and, therefore, increase yield. According to Qaim,^58^ GM crops have an estimate of 22% of yield advantage when compared to non-GM crops. For instance, if the EU were to adopt more GM crops, there could be a reduction of 33 million tons of CO_2_ equivalents per year,^59^ especially because EU imports come from countries with less strict environmental laws and EU trade agreements do not require imports to be produced sustainably. With an increasing demand for food, the producing countries tend to increase their land use, and therefore their GHG emissions. About 11 million hectares of land were deforested outside of the EU to grow crops to be consumed in the EU.^60^ Besides the benefits of HT and IR crops, the development and adoption of GM crops with abiotic stress tolerance have the potential to reduce crop damage in face of climate change and increase its yield.^59^

### 5.2. Impacts on Human Health

A great amount of attention is given to human nutrition, because food is crucial for human health and plays an important role in many diseases.^61^ Genetic modification is a tool that can improve the biofortification of crops, staple or non-staple. Golden Rice is an example of biofortification using genetic modification, targeting regions with vitamin A deficiency. Another example is GM wheat, biofortified to have greater bioavailability of zinc and iron, to prevent micronutrient deficiency; soybean also have been biofortified with different nutritional goals such as enhanced iron, carotenoid, sulfur-containing amino acids, protein content, oleic acid, and canthaxanthin (provitamin); sorghum was biofortified for its low digestibility, improved bioavailability of provitamin A, Zn, Fe, vitamin E, and tanin-free.^62–71^ Furthermore, biofortification for enhanced bioavailability of provitamin A has been done in tomato, potato, sweet potato, and cassava vegetables. Not only for provitamin A, but vegetables were also modified to have higher micronutrient profiles, greater carotenoid content in tomato, and higher folate content in lettuce, which deficiency is associated with cardiovascular diseases and cancer in humans.^71–74^ Some fruits were also modified for biofortification, which is the case of banana with enhanced provitamin A contents, apple with more flavonoids, and changes in anthocyanin content in strawberry.^71,75–77^ Beyond being more nutritious, GM food can also be safer than their non-GM counterparts. Besides going through greater RAs for allergenicity and toxicity, GM maize resistant to insect pests can, for example, have lower mycotoxin concentration when compared to non-GM maize, as insects assist fungus spores’ migration and colonization.^78,79^ A study performed by Myskja^80^ showed that people are less likely to oppose GM crops if they have enhanced nutrition values than increased yield traits, because consumers do not see the direct impact of increased yield in human health, so they assume that there are none.^79^

Before the adoption of GMHT crops, a broad range of herbicides was used. GMHT crops allowed broader and less persistent herbicide to be used. This adoption has shown a decline in the use of herbicides, and a reduction in the environmental impact of pesticide use.^81^ Glyphosate, which is the most used herbicide on GMHT crops, does not bioaccumulate in organisms because of its high solubility in water. It is decomposed quickly by microorganisms when compared to other herbicides, making its half-life shorter in soil and water, decreasing chances of animal and human contamination. Besides, in the soil, this herbicide is virtually inactive biologically.^82^

## 6. Conclusions

Food biotechnology is greatly important to ensure food security in face of climate change and growing demand for food. Since developing countries are often the most severely affected by climate changes, the adoption of GM crops could help sustain agricultural productivity and yield under adverse environmental and social conditions. The largest producing countries of agricultural commodities could also benefit from GM adoption if this had not been done yet.

Opinion-related decisions about the adoption of GMOs cause low technology adoption, which impacts countries’ capacity to adjust to evolving climate conditions, compete in global markets, and deliver more benefits to society. On the other hand, if the decision to approve GM events is based on scientific-related evidence, public trust in new technologies can be strengthened, facilitating responsible innovation in agriculture.

Having one global RA for widely cultivated crops such as maize, cotton, soybean, and canola, and common traits such as insect resistance and herbicide tolerance would ensure fewer scarce resources are spent on regulation processes that all have the same content, facilitating the adoption of those crops, especially in developing countries. Novel traits with new proteins should continue to undergo specific RAs, but countries could streamline their approval process by recognizing completed assessments from other jurisdictions and adapting them to their own national variables. For 30 years, GM crops have consistently demonstrated higher yields and consumption safety, providing a valued solution for food insecure countries. To enable the adoption of higher yielding crop varieties, food insecure and resource constrained countries can benefit by the safety confirmation of the thousands of GM crop RAs that have been completed.

## Data Availability

The data utilized in this research is available at https://www.isaaa.org/gmapprovaldatabase/default.asp.
